# Survival in hematological malignancies in the Nordic countries through a half century with correlation to treatment

**DOI:** 10.1038/s41375-023-01852-w

**Published:** 2023-02-24

**Authors:** Kari Hemminki, Janne Hemminki, Asta Försti, Amit Sud

**Affiliations:** 1 Biomedical Center, Faculty of Medicine and Biomedical Center in Pilsen, Charles University in Prague, 30605 Pilsen, Czech Republic; 2 Division of Cancer Epidemiology, German Cancer Research Center (DKFZ), Im Neuenheimer Feld 580, D-69120 Heidelberg, Germany; 3 Hopp Children’s Cancer Center (KiTZ), Heidelberg, Germany; 4 Division of Pediatric Neurooncology, German Cancer Research Center (DKFZ), German Cancer Consortium (DKTK), Heidelberg, Germany; 5 Division of Genetics and Epidemiology, The Institute of Cancer Research, London, UK; 6 Haemato-oncology Unit, The Royal Marsden Hospital NHS Foundation Trust, Sutton, UK

**Keywords:** Risk factors, Medical research

## Abstract

Studies of survival in hematological malignancies (HMs) have generally shown an improvement over time, although most of these studies are limited by a short follow-up period. Using the NORDCAN database with data from Denmark, Finland, Norway and Sweden, we follow periodic increases in relative survival in seven HMs through half a century up to 2015–2019. Five-year survival improved in all seven HMs, reaching 90% for Hodgkin lymphoma (HL), myeloproliferative neoplasias and chronic lymphocytic leukemia (CLL), 60% for multiple myeloma (MM) and chronic myeloid leukemias (CMLs), 50% for the myelodysplastic syndromes and 30% for acute myeloid leukemia (AML). Improvements in survival over 50 years ranged from 20% to more than 50% units across the different HMs. The likely reasons for such progress include earlier diagnoses, improved risk stratification and advances in treatment. We observed differing temporal trends in improvements in survival. The gradual increases observed in HL, CLL and AML highlight the impact of optimization of existing therapies and improvements in diagnostics and risk stratification, whereas the rapid increases observed in the CMLs and MM highlight the impact of novel therapies. Recent therapeutic advances may further improve survival in HMs where survival remains low such as in AML.

## Introduction

The major aim of oncology is improving the quality of life and survival of patients whilst minimizing treatment-related toxicity. In hematological malignancies (HMs), there is a reliance on the administration of systemic anti-cancer therapies to achieve this goal. The different systemic treatment modalities now used to treat many cancer types were first pioneered in the HMs. These include systemic chemotherapy (e.g., vinca alkaloids in Hodgkin lymphoma (HL) and acute lymphoblastic leukemia (ALL)), targeted therapies (e.g., tyrosine kinase inhibitors in chronic myeloid leukemia (CML), hematopoietic stem cell transplantation (HSCT, e.g., autologous and allogenic stem cell transplantation in multiple myeloma (MM) and the leukemias), monoclonal antibodies (e.g., rituximab in B-cell non-Hodgkin lymphomas (NHL)) and immunotherapies (e.g., CAR-T-cell therapy in ALL) [1–5].

The development of these therapies, as well as to optimization of existing therapies and supportive care, has resulted in improvements in survival in the HMs in economically developed countries [6–8]. However, a limitation in existing HM survival data is its relatively recent duration and case selection of specific populations. The Nordic cancer registries are a powerful resource as they are the oldest cancer registries in the world and have almost complete case ascertainment, allowing for the study of cancer survival over 50 years with minimization of bias introduced through ascertainment [9]. Grouped data from these registries are accessible as the NORDCAN database, which has been the source of numerous survival studies, including those on HMs starting from the 1960s [10–12].

Here, we use the NORDCAN database to analyze survival in all available specific HMs from Denmark (DK), Finland (FI), Norway (NO) and Sweden (SE). We follow the periodic increases in survival and try to match these with known changes in the diagnosis and treatment of HMs. The organization of healthcare is largely similar in these countries offering widespread access to the population. However, economic resources differ between the countries. As a comparator, healthcare expenditure per capita in the year 2000 was $2496 (8.8% of GNP) in DK, $1723 (7.1%) in FI, $2949 (7.7%) in NO and $2173 (7.3%) in SE (www.macrotrends.net). During the period from 1970 to 2019, demographic changes have taken place and life expectancy has increased in FI by 11.6 years and in other countries by 8 years. We show data on 1-year and 5-year relative survival between 1970–1974 and 2015–2019 and the survival difference between these periods.

## Methods

The data originate from the NORDCAN database which is a compilation of data from the Nordic cancer registries as described [9, 13]. The database was accessed at the IARC website (https://nordcan.iarc.fr/en/database#bloc2). The analysis included specific HMs, defined by the International Classification of Diseases (ICD) version 10 codes: NHL C82-86, HL C81, MM C90, myelodysplastic syndrome (MDS) D46, myeloproliferative disease (MPN) D45+D47.1,3-5, chronic lymphatic leukemia (CLL) C91.1, acute myeloid leukemia (AML) C92.0+C93.0+C94.0+C94.2+C94.4-5 and the CMLs C92.1+C93.1+C94.1. The ICD-10 classification does not distinguish disease subtypes that exist for each HM. Moreover, C93.1 (chronic myelomonocytic leukemia, CMML) is now recognized to belong to the MDS/MPN HMs) [14]. Data for unspecified HMs were not considered. NHL was included only in the first tabulation as a reference, as it was not possible to distinguish between specific subtypes with large survival differences. Data for ALL were not included as it was not possible to distinguish childhood and adult disease.

Survival data were available from 1970 through 2019 and the analysis was based on the cohort survival method for periods from 1970 to 2014 and a hybrid analysis combining period and cohort survival in the last period 2015–2019, as previously detailed [13, 15]. Age-standardized relative survival was estimated using the Pohar Perme estimator [16]. Age-standardization was performed by weighting individual observations using external weights as defined on the IARC website [17]. National general population life-tables stratified by sex, year and age were used in the calculation of expected survival. Death certificate only cases were not included. Patients 90 years or older were excluded. Groups were analyzed if minimum 30 patients were alive at the start and with minimum 3 patients in any one of age groups used for weights. Periodic 5-year survival data were plotted for the seven HMs. The underlying data were tabulated in 5-year intervals for common HMs and in 10-year intervals in rarer HMs. In the tabulations, 95% confidence intervals (CIs) were included and significant periodic increases (defined as non-overlapping 95% CIs) were indicated with an asterisk. Age-specific survival data were not available in the current NORDCAN release and these data were obtained from an earlier version of NORDCAN, extending follow-up to the end of 2016.

We also calculated the difference in relative survival percentage between year 1 and year 5 of the early (1970–1979) and late (2010–2019) time periods [18]. A small difference indicates favorable survival between years 1 and 5 after diagnosis. Smoothing was used for graphical representation.

Information on the treatments applied for subtypes of HMs was collected from the relevant publications, marketing authorization documents of the Swedish Medical Products Agency or from the European Medicines Agency approval dates.

## Results

In Table 1 we show recent data (2012–2016) on overall 5-year survival and survival in the age group 70–89, with the underlying case numbers and age-standardized (world) incidence for men and women in the four countries. Greater than 50% of HMs, excluding cases of HL and MPNs, occur in this older age group. However, relative survival was markedly lower in these older patients and with particularly low relative survival in AML, MM and MDS. Incidence rates are similar between the Nordic countries with the exception of a higher incidence of CLL, MDS and MPN in DK. Male incidence rates were generally higher than female rates and the difference was about 2-fold for CML and somewhat less for MDS.

**Table 1:** Relative 5-year survival (%), case numbers and age-standardized incidence (world)/100,000 for all patients and for the aged (diagnosed at age 70–89 years), 2012–2016.

	Denmark		Finland		Norway		Sweden	
	All	Old	All	Old	All	Old	All	Old
*Men*								
Acute myeloid leukemia, survival, %	22	2	19	1	19	1	27	1
Cases	506	267	482	246	405	185	828	392
Incidence	1.9		2.0		2.0		2.0	
Chronic lymphatic leukemia, survival, %	84	64	77	44	85	60	81	53
Cases	1494	750	922	500	993	475	1855	1030
Incidence	5.2		3.2		4.2		3.6	
Chronic myeloid leukemia, survival, %	56	35	58	23	53	22	64	35
Cases	313	147	177	74	257	122	521	239
Incidence	1.3		0.8		1.2		1.2	
Hodgkin lymphoma, survival, %	89	66	86	39	84	28	86	43
Cases	447	66	474	69	435	45	615	111
Incidence	2.8		3.0		3.0		2.2	
Multiple myeloma, survival, %	55	25	43	16	49	24	54	20
Cases	1279	675	1057	578	1190	651	2053	1126
Incidence	4.4		3.7		4.9		4.0	
Myelodysplastic syndromes, survival, %	42	25	15	1	37	14	42	24
Cases	887	593	407	328	500	358	951	664
Incidence	2.9		1.2		1.8		1.6	
Myeloproliferative diseases; survival, %	75	46	69	36	80	45	–	–
Cases	1396	586	762	368	726	302	1307	601
Incidence	5.4		2.9		3.3		2.9	
*Women*								
Acute myeloid leukemia, survival, %	22	–	26	2	20	–	25	2
Cases	456	216	442	228	361	150	782	412
Incidence	1.8		1.5		1.8		1.7	
Chronic lymphatic leukemia, survival, %	91	81	82	55	92	70	88	73
Cases	904	500	611	388	679	404	1141	700
Incidence	2.7		1.5		2.4		1.9	
Chronic myeloid leukemia, survival, %	67	20	63	26	61	12	66	38
Cases	207	104	121	55	165	81	363	155
Incidence	0.8		0.5		0.7		0.8	
Hodgkin lymphoma, survival, %	87	48	89	58	88	45	89	59
Cases	307	53	366	71	306	47	507	109
Incidence	2.1		2.5		2.2		1.9	
Multiple myeloma, survival, %	58	37	46	17	51	23	53	20
Cases	981	556	1049	623	975	559	1497	884
Incidence	3.0		2.9		3.5		2.6	
Myelodysplastic syndromes, survival, %	51	31	24	9	43	25	47	35
Cases	549	372	358	291	327	230	650	72
Incidence	1.6		0.7		1.0		1.0	
Myeloproliferative diseases; survival, %	88	70	78	55	91	73	–	–
Cases	1544	797	934	536	823	407	1397	728
Incidence	5.3		2.9		3.3		2.7	

**Fig. 1:**
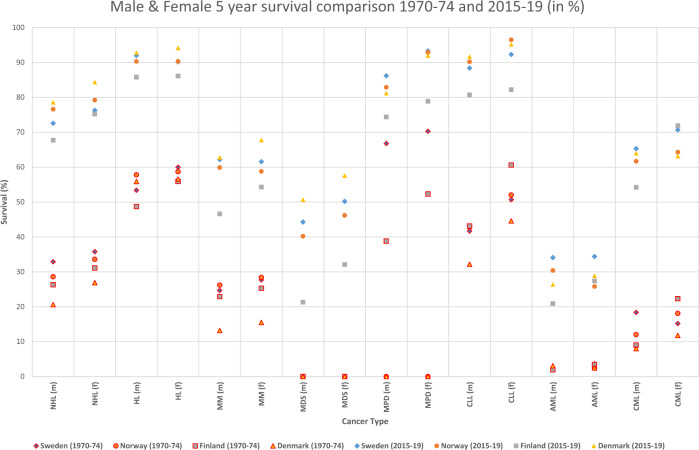
Relative 5-year survival in hematological malignancies in the Nordic countries in 1970–1974 and 2015–2019 based on the NORDCAN database. Note that the 1970–1974 datapoints were missing for MDS and for MPNs in DK and NO, and the symbols are marked as 0.

Five-year relative survival in 1970–1974 and 2015–2019 for men and women in each Nordic country is shown in Fig. 1. Male and female survival data appeared similar. The largest improvements were observed for NHL (from 30 to 80%), for CLL in men (40 to 90%) and for CMLs (from 15 to 65%). For most other HMs the improvement varied between 20 and 30% units. Country-specific differences were small for NHL and HL but were larger for other HMs (note that early datapoints were missing for MDS and for MPNs in DK and NO). For many of the HMs, relative survival was lowest in DK in 1970–1974. However, in 2015–2019 relative survival in DK was the highest and FI tended to be the lowest.

To assess the change in survival over the 50-year period from 1970 to 2019, we collated relative survival in the first and the last 10-year period in Table 2 by sex and country. We use the ratios of survival between 2010–2019 and 1970–1979 to assess the temporal trend in survival. The relative survival ratios were larger for 5-year compared to 1-year relative survival. The ratios were below 2 for 1-year relative survival for all HMs, except for AML ranging up to 3. The ratios for 5-year relative survival were below 2 for HL, MDS and MPN; they were slightly higher for CLL, and reached 4 for MM and CML, and up to 8 for AML.

**Table 2:** Improvements (ratios) in and the differences between 1- and 5-year survival in hematological malignancies between 1970–1979 and 2010–2019.

	Men								Women							
	Denmark		Finland		Norway		Sweden		Denmark		Finland		Norway		Sweden	
		Diff		Diff		Diff		Diff		Diff		Diff		Diff		Diff
*HL*	1-year															
1970–1979	78.0 [75.0–80.7]		77.4 [74.2–80.3]		75.7 [72.4–78.6]		76.5 [74.3–78.5]		79.3 [75.7–82.4]		80.0 [76.4–83.1]		80.1 [76.3–83.3]		78.1 [75.6–80.4]	
2010–2019	**95.9 [94.3–97.0]**		93.1 [91.4–94.6]		94.5 [92.7–95.8]		94.9 [93.5–96.0]		94.6 [92.4–96.2]		93.7 [91.3–95.4]		**95.3 [93.1–96.8]**		95.0 [93.3–96.3]	
Ratio	1.23		1.2		1.25		1.24		1.19		1.17		1.19		1.22	
	5-year															
1970–1979	58.4 [54.7–61.9]	19.6	54.2 [50.2–58.0]	23	57.2 [53.1–61.1]	19	56.2 [53.4–58.9]	20.3	61.1 [56.7–65.2]	18.1	62.9 [58.4–67.0]	17.1	64.5 [59.8–68.8]	15.6	62.6 [59.2–65.9]	15.5
2010–2019	**92.0 [89.4–94.0]**	3.9	88.1 [85.2–90.4]	5	89.1 [86.3–91.3]	5.4	89.7 [87.3–91.6]	5.2	**91.3 [87.8–93.7]**	3.3	88.3 [84.6–91.2]	5.4	88.8 [85.0–91.7]	6.5	89.4 [86.4–91.7]	5.6
Ratio	1.58		1.63		1.56		1.6		1.49		1.4		1.29		1.27	
*MM*	1-year															
1970–1979	47.4 [44.1–50.6]		62.9 [58.9–66.7]		64.3 [61.4–67.1]		64.6 [62.5–66.7]		52.8 [49.2–56.3]		64.5 [61.1–67.8]		72.1 [69.2–74.9]		69.6 [67.3–71.7]	
2010–2019	86.7 [85.1–88.0]		79.7 [77.7–81.5]		85.3 [83.6–86.8]		**88.6 [87.5–89.6]**		**89.4 [87.8–90.8]**		83.5 [81.6–85.1]		86.1 [84.3–87.7]		88.1 [86.8–89.3]	
Ratio	1.83		1.27		1.33		1.38		1.69		1.29		1.19		1.27	
	5-year															
1970–1979	15.0 [12.5–17.7]	32.4	29.0 [24.8–33.3]	34	26.4 [23.5–29.3]	31	27.3 [25.1–29.6]	37.3	19.1 [16.2–22.3]	34	27.3 [23.9–30.7]	37.2	29.3 [26.2–32.4]	43	29.4 [27.0–31.7]	40.2
2010–2019	**60.5 [57.6–63.3]**	31.5	45.9 [42.8–48.9]	34	57.2 [54.1–60.2]	32	59.6 [57.4–61.8]	29	**65.1** **[61.9–68.2]**	24	50.4 [47.4–53.3]	33.1	56.3 [52.9–59.5]	30	57.5 [55.0–60.0]	30.6
Ratio	4.03		1.58		2.17		2.18		3.41		1.85		1.92		1.96	
*MDS*	1-year															
1980–1989	72.7 [58.8–82.6]		–		–		59.2 [50.3–67.1]		61.8 [45.2–74.6]		–		–		61.8 [51.8–70.3]	
2010–2019	**80.6 [78.3–82.7]**		68.6 [64.0–72.8]		77.3 [74.0–80.3]		78.6 [76.1–80.8]		**85.2 [82.6–87.4]**		71.8 [67.0–76.0]		73.0 [68.4–77.0]		81.6 [78.8–84.1]	
Ratio	1.11						1.33		1.38						1.32	
	5-year															
1980–1989	49.3 [34.0–62.9]	23.4	–		–		29.8 [21.6–38.4]	29.4	38.7 [22.3–54.8]	23	–		–		28.4 [19.8–37.5]	33.4
2010–2019	**48.5 [44.9–52.1]**	32.1	21.5 [16.4–27.1]	44	38.0 [33.1–42.9]	39	43.6 [40.1–47.0]	35	**57.1 [52.7–61.2]**	28	29.7 [24.2–35.5]	42.1	43.3 [37.3–49.1]	30	47.3 [43.0–51.4]	34.3
Ratio	0.98						1.46		1.48						1.67	
*MPD*	1-year															
1970–1979	–		72.9 [66.7–78.2]		–		83.0 [79.6–85.9]		–		78.2 [73.1–82.4]		–		88.6 [85.6–91.0]	
2010–2019	95.8 [94.7–96.7]		91.8 [89.8–93.4]		95.7 [94.1–96.9]		**96.7 [95.6–97.5]**		**99.1 [98.2–99.6]**		94.3 [92.8–95.5]		97.4 [96.0–98.3]		98.1 [97.2–98.7]	
Ratio			1.26				1.17				1.21				1.11	
	5-year															
1970–1979	–		50.2 [42.3–57.5]	23	–		63.9 [58.7–68.7]	19.1	–		55.0 [48.5–61.1]	23.2	–		70.0 [65.3–74.2]	18.6
2010–2019	80.7 [77.8–83.3]	15.1	73.7 [69.3–77.5]	18	82.4 [78.1–85.9]	13	**85.1 [82.3–87.4]**	11.6	91.0 [88.5–92.9]	8.1	77.1 [73.6–80.2]	17.2	90.7 [87.1–93.3]	6.7	**92.7 [90.3–94.5]**	5.4
Ratio			1.47				1.33				1.4				1.32	
*CLL*	1-year															
1970–1979	65.5 [62.7–68.2]		77.4 [73.7–80.6]		75.4 [71.5–78.9]		78.6 [76.0–80.9]		71.5 [67.9–74.8]		86.9 [83.5–89.7]		76.2 [71.1–80.5]		82.8 [79.6–85.5]	
2010–2019	**98.1 [97.1–98.8]**		94.5 [93.0–95.7]		97.6 [96.3–98.4]		97.0 [96.2–97.7]		**98.7 [97.5–99.4]**		94.3 [92.6–95.7]		98.4 [97.0–99.2]		98.4 [97.5–99.0]	
Ratio	1.5		1.22		1.29		1.23		1.38		1.09		1.29		1.19	
	5-year															
1970–1979	33.2 [30.0–36.4]	32.3	49.4 [44.3–54.2]	28	41.3 [36.4–46.1]	34	44.7 [41.2–48.1]	33.9	43.6 [39.2–48.0]	28	62.0 [56.6–67.0]	24.9	51.4 [44.9–57.5]	25	57.1 [52.7–61.4]	25.7
2010–2019	**89.9 [87.0–92.2]**	8.2	79.9 [76.4–82.9]	15	88.9 [85.6–91.5]	8.7	87.0 [84.8–88.9]	10	**94.8** **[91.5–96.8]**	3.9	81.0 [76.9–84.4]	13.3	93.3 [89.6–95.7]	5.1	91.6 [89.1–93.5]	6.8
Ratio	2.71		1.62		2.15		1.95		2.17		1.31		1.82		1.6	
*AML*	1-year															
1970–1979	17.1 [14.5–19.9]		19.4 [15.6–23.5]		21.5 [18.2–25.1]		16.8 [14.1–19.7]		18.0 [15.2–20.9]		16.6 [13.5–20.1]		18.3 [15.1–21.8]		20.7 [17.7–23.9]	
2010–2019	50.2 [47.0–53.3]		48.3 [44.7–51.8]		47.9 [44.3–51.4]		**52.8 [50.2–55.4]**		52.8 [49.1–56.2]		52.1 [48.5–55.6]		45.4 [41.4–49.4]		**52.9 [50.2–55.5]**	
Ratio	2.94		2.49		2.23		3.14		2.93		3.14		2.48		2.56	
	5-year															
1970–1979	3.3 [2.1–5.0]	13.8	1.9 [0.90–3.6]	18	4.0 [2.5–6.0]	18	2.9 [1.9–4.4]	13.9	4.0 [2.6–5.7]	14	3.6 [2.2–5.5]	13	3.5 [2.2–5.2]	15	3.7 [2.4–5.5]	17
2010–2019	25.9 [22.6–29.2]	24.4	22.3 [19.0–25.8]	26	26.9 [23.3–30.6]	21	**33.2 [30.5–36.0]**	19.6	28.6 [24.9–32.5]	24	25.7 [22.2–29.4]	26.4	27.2 [23.1–31.5]	18	**32.0 [29.2–34.9]**	20.9
Ratio	7.85		11.74		6.73		11.45		7.15		7.14		7.77		8.65	
*CML*	1-year															
1970–1979	43.7 [38.3–48.9]		61.1 [53.3–68.1]		61.1 [54.0–67.4]		60.0 [54.3–65.2]		52.9 [47.0–58.5]		70.3 [63.3–76.2]		62.5 [54.9–69.2]		59.9 [53.7–65.5]	
2010–2019	86.0 [82.7–88.8]		79.7 [74.3–84.0]		86.0 [82.0–89.1]		**87.3 [84.6–89.5]**		88.7 [84.8–91.6]		88.9 [83.5–92.5]		85.0 [79.9–88.9]		**89.0 [86.0–91.4]**	
Ratio	1.97		1.3		1.41		1.46		1.68		1.26		1.36		1.49	
	5-year															
1970–1979	11.2 [7.8–15.1]	32.5	17.5 [11.7–24.2]	44	14.1 [9.6–19.5]	47	19.2 [14.9–23.9]	40.8	13.6 [9.9–18.0]	39	23.0 [17.3–29.2]	47.3	21.4 [15.3–28.1]	41	16.8 [12.4–21.7]	43.1
2010–2019	63.7 [57.7–69.1]	22.3	52.6 [44.6–60.1]	27	59.3 [52.1–65.9]	27	**65.6 [60.9–69.9]**	13.7	63.1 [56.3–69.1]	26	66.1 [56.0–74.4]	22.8	63.6 [55.2–70.9]	21	**68.6 [63.2–73.5]**	20.4
Ratio	5.69		3.01		4.21		3.42		4.64		2.87		2.97		4.08	

Improvement (ratio) = survival in 2010–2019 divided by survival in 1970–1979.

Diff = difference between 1- and 5-year survival (% units) in the first and the last 10-year period.

Bold values show the highest survival for men and women in 2010–2019.

In addition to the ratios of relative survival, we calculated the difference between 1- and 5-year survival in the early (1970–1979) and late (2010–2019) time periods (Table 2). For the majority of HMs the difference between survival was greater in 1970–1979 when compared to 2010–2019. This was most marked in HL where the difference was 20% units in 1970–1979 and 5% units in 2010–2019. Exceptionally for AML and MDS, we noted an increase in the difference in survival from 1970–1979 to 2010–2019.

**Fig. 2:**
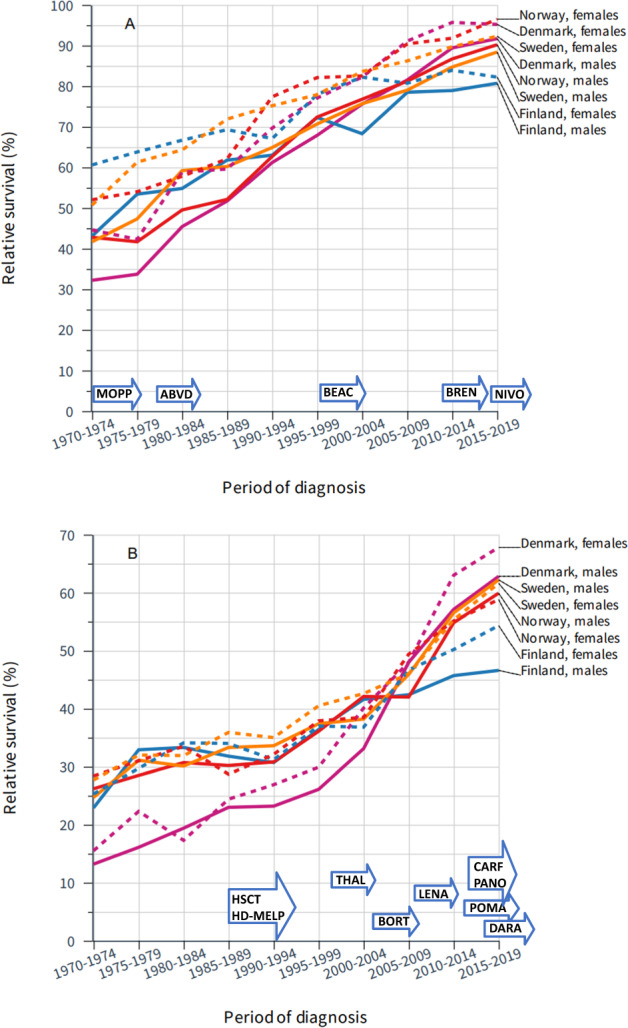
Survival trends for Hodgkin lymphoma and multiple myeloma in the Nordic countries. Relative 5-year survival in Hodgkin lymphoma (**A**) and multiple myeloma (**B**) in the Nordic countries from 1970 to 2019. The underlying data are available in Supplementary Table 1 with 95% CIs allowing assessment of significant improvements between subsequent periods. The introduction of novel therapies is shown on top of *x*-axis with details in Discussion. **A** MOPP nitrogen mustard, vincristine, procarbazide and prednisone, ABVD adriamycin, bleomycin, vinblastine, dacarbazine, BEAC (BEACOPP) bleomycin, etoposide, adriamycine, cyclophosphamide, vincristine, procarbazine, and prednisone, BREN Brentuximab, NIVO Nivolumab. **B** HSCT hematopoietic stem cell transplantation, HD-MELP high-dose melphalan, THAL thalodomine, BORT bortezomib, LENA lenalidomide, POMA pomalidomide, PANO panobinostat, CARF carfilzomib, DARA daratumumab.

Relative 5-year survival for HL and MM is shown Fig. 2A, B and the underlying data are tabulated in Supplementary Table 1. Five-year relative survival in HL in 1970–1974 was over 60% for women and somewhat less for men and reached approximately 90% for both sexes in 2015–2019. Country-specific differences were generally small. Periodic 10-year survival figures increased monotonically (increase in each 10-year period without exception) with decreasing steps for men and women (Supplementary Table 1). For MM, the starting 5-year relative survival was lower than that of HL and improvement was slow until 2000, where a marked increase was noted; significant periodic improvements in 5-year survival took place after the year 2000 (asterisk in Supplementary Table 1). Whilst relative survival in DK for MM started lower than that of other countries, by the end of the study period, it exceeded that of other countries. Relative survival of MM in FI was lower than that of other countries in the last time period (Supplementary Table 1).

**Fig. 3:**
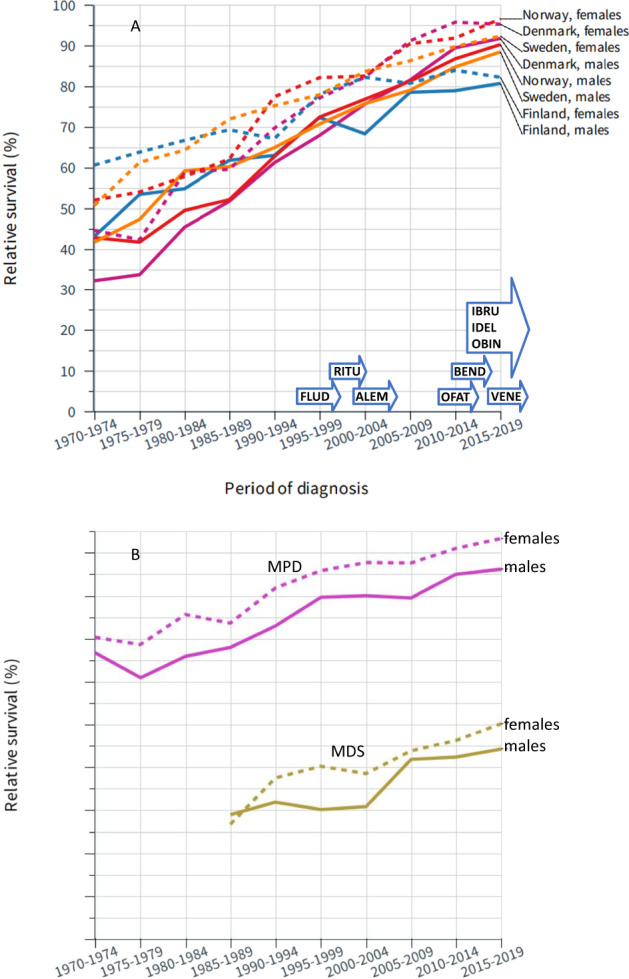
Survival trends for chronic lymphoid leukemia, myelodyplastic syndrome and myeloproliferative disease in the Nordic countries. Relative 5-year survival in chronic lymphoid leukemia (**A**) and myelodysplastic syndrome and myeloproliferative disease (**B**) in the Nordic countries from 1970 to 2019. The underlying data are available in Supplementary Table 1 with 95% CIs allowing assessment of significant improvements between subsequent periods. The introduction of novel therapies is shown on top of *x*-axis with details in Discussion. **A** FLUD fludarabine, RITU rituximab, ALEM alemtuzumab, OFAT ofatumumab, BEND bendamustine, OBIN obinutuzumab, IDEL idelalisib, IBRU ibrutinib, VENA venetoclax.

Relative 5-year survival in CLL demonstrated a consistent improvement over the 50-year period (Fig. 3A and Supplementary Table 1). The increase was close to linear, and for NO and SE, survival in each 5-year period demonstrated a consistent increase. The 5-year relative survival reached 95% in DK and NO women, but just exceeded 80% in FI men and women. For MDS and MPN the SE data were most complete and were therefore plotted in Fig. 3B; however, all available data are shown in Supplementary Table 1. For SE and DK, 5-year relative survival in MDS increased to over 40% (DK women 57%), but survival in NO and FI was lower. Survival in MPN was far better than that in MDS, and female 5-survival reached over 90% and male survival was over 80% (FI men and women below 80%).

**Fig. 4:**
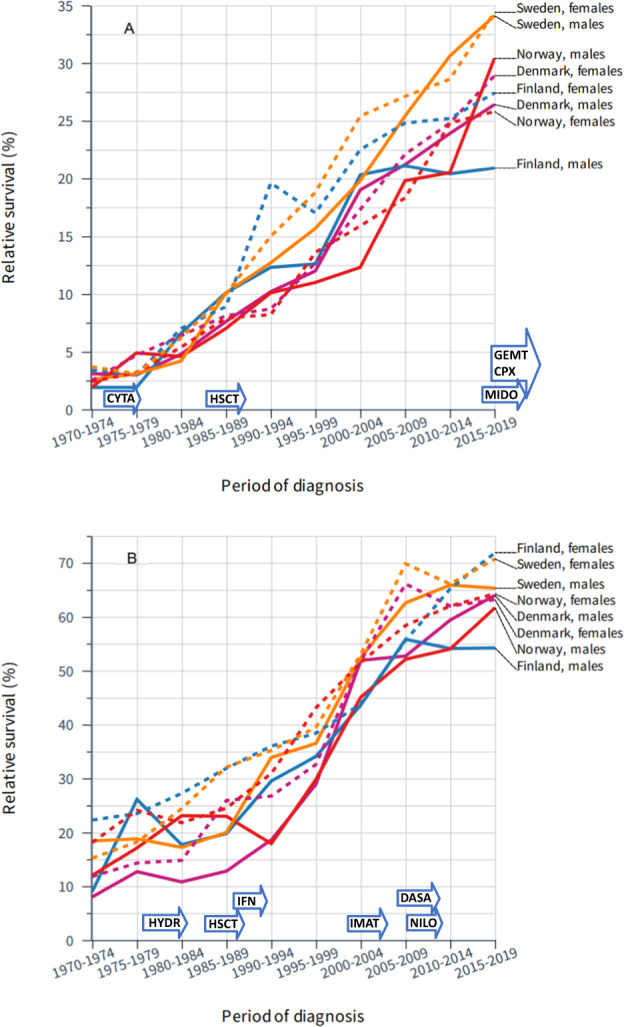
Survival trends for acute myeloid leukemia and chronic myeloid leukemia in the Nordic countries. Relative 5-year survival in acute myeloid leukemia (**A**) and chronic myeloid leukemia (**B**) in the Nordic countries from 1970 to 2019. The underlying data are available in Supplementary Table 1 with 95% CIs allowing assessment of significant improvements between subsequent periods. The introduction of novel therapies is shown on top of *x*-axis with details in Discussion. **A** CYTA cytarabine+daunorubicin, HSCT hematopoietic stem cell transplantation, CPX CXP-351, GEMT gemtuzumab, MIDO midostaurin. **B** HYDR hydroxyurea, HSCT hematopoietic stem cell transplantation, IFN interferon alfa, IMAT imatinib mesylate, NILO nilotinib, DASA dasatinib.

Survival in AML was markedly low in the 1970s, with a 1-year relative survival of approximately 20% and 5-year survival <5% (Fig. 4A and Supplementary Table 1). However, constant improvement took place and in SE, patients with AML reached a 5-year relative survival of 34% in 2015–2019. For the other groups, survival varied between 25 and 30% but in FI men it was barely over 20% with little improvement since 2000. Survival in CML has been much better than that for AML, with significant improvements noted since 1990 (Fig. 4B).

Table 2 shows the highest survival rate for each HM in each Nordic country (emboldened) by sex for the most recent 1- and 5-year survival period. DK had the largest number of HMs with the highest 1-year relative survival (3 male and 4 female), followed by SE (4 male and 2 female) and NO (1 female). DK had the largest number of HMs with the highest 5-year survival (4 male and 4 female) followed by SE (3 male and 3 female). Survival percentages differed minimally between countries, but there was some consistency, as the country with the highest relative survival for a given disease was most often sex-concordant.

## Discussion

This is a unique survival study of HMs capturing high-quality data from four countries spanning half a century. To achieve such an analysis, there are a number of compromises to ensure the results are robust and meaningful. Firstly, although all main HM entities contain clinically distinct subgroups, we have omitted NHL and ALL due to the well-recognized heterogeneity in disease characteristics in different subgroups and age groups. Secondly, time-dependent diagnostic drifts are possible for HMs although a SE study, including NHL, HL, MM and CLL over years 1964 through 2003, found that 97.9% of the reported tumors fulfilled the diagnostic criteria and the completeness of the cancer registry data varied between 95 and 99% [19]. However, the classification of MDS and many types of MPNs (and reporting to cancer registries) has evolved since the introduction of 2001 WHO classification of HMs [14]. We therefore report data on MDS and MPN in Fig. 3B only for SE where data collection has been stable over decades; for example, high familial risks were reported for polycythemia vera from 1975 onwards which is only possible when diagnostic data are reasonably reliable [20]. The refined diagnostic criteria have allowed for the development of defined treatment guidelines for entities such as CML [21]. Unfortunately, NORDCAN includes CMML among the codes of CML, instead of grouping it in MDS/MPN [14]. The incidence of CMML has been estimated at 0.57/100,000 in men and 0.25/100,000 in women (European age standard) in Switzerland (and in the USA) [22]. Our CML incidence figures from Table 1 (1.1/100,000 for men 0.7/100,000 for women, world standard) would translate to 1.6/100,000 for men and 1.1/100,000 using the European standard population. Thus, in the NORDCAN CML population, about 1/3 of men and 1/4 of women may be CMML patients. Relative 5-year survival in CMML, according to the above Swiss/USA study, was about 25% which was much below the present 55–65% survival for CML (Table 1) implying that survival in CML was underestimated in the NORDCAN data because of CMML contamination.

From 2000, the Nordic countries were included in the set of European collaborative studies covering 11 types of HMs for over a decade [6, 7]. For most HM types, the Nordic countries showed the best survival but essentially all countries were able to improve survival within the short observation period. The survival results showed strong age-dependence for almost all HMs. In the present study, we could confirm the positive survival development and the age-dependence for the seven types of HMs, and could extend the observation period for a half century up to the year 2019. A number of factors are likely to explain the progress: centralization of care, earlier diagnoses, enhanced risk stratification, including more sensitive disease detection, the use of novel therapies, optimization of existing treatments and better supportive care. During this study period, a wide range of technological advances has been made that have transformed the diagnosis and management of HMs. These include new imaging modalities (e.g., computed tomography, magnetic resonance imaging, positron emission tomography), flow cytometry, chromosomal techniques (e.g., cytogenetics and fluorescence in situ hybridization) and techniques in molecular biology (e.g., Sanger sequencing and high-throughput sequencing) [23–25]. This 50-year time series showed notable HM-specific temporal features, and below we consider the possible contributing factors to improvements in survival. We are aware of the recent introduction of numerous novel therapies in hematology practice in the form of immunotherapy and small molecule inhibitors which may not be captured in the 5-year survival in the last time period (2015–2019) in NORDCAN because the survival method generates the data for the last 5-year period by comparing to the previous period [26].

For HL and CLL, 5-year survival increased throughout the 50-year period; for CLL the increase was approximately linear but for HL the rate of improvement plateaued in later time periods. For both of these diseases, the difference between 1- and 5-year relative survival decreased over time (Table 2) indicating progress in care also improved longer-term survival. HL treatment traditionally has been based on multi-drug chemotherapy (marked as MOPP, nitrogen mustard, vincristine, procarbazide and prednisone and ABVD, adriamycin, bleomycin, vinblastine, dacarbazine in Fig. 2) and radiotherapy [27]. Later, the BEACOPP (bleomycin, etoposide, adriamycine, cyclophosphamide, vincristine, procarbazine, and prednisone) regimen was developed to improve treatment results [28]. The treatment regimens based on risk stratification have been introduced to reduce rates of long-term complications whilst maintaining a high cure rate. Novel agents such as drug-antibody conjugates and checkpoint inhibitors (Brentuxmab and Nivolumab in Fig. 2) have offered treatment options for individuals refractory to first-line combination chemotherapy [27]. In CLL, the addition of rituximab to purine analogs and alkylating chemotherapy became a standard of care in the early 2000s. Another monoclonal antibody, alemtuzumab was reserved for patients with the chromosomal defect del(17p) as they respond poorly to chemotherapy [29]. More recently, novel therapies such as PI3Kδ, BTK and BCL2 inhibitors have transformed the management of patients with CLL, although their impact on survival may only be seen in the later years of this study [30]. Despite advances in therapies, a watch-and-wait strategy remains the standard of care for early-stage CLL and approximately 30% of cases will never require treatment for their CLL. As we consider survival in all CLL patients (treated and not treated), the positive effects of improved treatments will be diluted when analyzing relative survival in all cases.

AML is distinct due to the low 5-year survival in the 1970s. Traditional intensive chemotherapy approaches incorporating anthracyclines and cytosine arabinosides (CYTA in Fig. 4) have remained the mainstay of treatment for the majority of AML cases during the study period and HSCT has been used in patients younger than 70 years [31, 32]. More accurate risk stratification, enhanced disease detection and improved supportive care including infection prophylaxis have likely led to the constant improvement in relative survival till 2015. However, since 2017, there has been an unprecedented growth in the number of approved therapies, such as monoclonal antibodies and FLT3, IDH1, IDH2, and BCL2 inhibitors, which, along with the above advances, has likely led to recent increases in 1-year relative survival [33]. However, the prognosis in older patients has remained poor (1 or 2% 5-year survival) and represents an area of unmet need [34].

The third group of HMs included MDS and MPN for which progress was observed throughout the observation period but data were somewhat limited for countries other than SE. The progress we observed in MDS was driven by 1-year survival. It is possible that diagnostic classification was not consistent in all countries as we observed large fluctuations in survival rates. MDS is a clonal bone marrow stem cell disorder resulting in impaired hematopoiesis which may be secondary to cancer treatment (chemotherapy or radiotherapy) [35]. Patients suffer from cytopenias which may be responsive to hematopoietic growth factors; lenalidomide (for lower-risk transfusion-dependent MDS with del(5q)), while high-risk patients may receive hypomethylating agents and allogeneic HSCT [35]. Even though 5-year survival reached 57% in DK women, it was lower in the other groups and in FI men it was only 21%. MPNs are disorders of the hematopoietic system that include myelofibrosis, polycythemia vera, and essential thrombocythemia, collectively known as Philadelphia chromosome–negative MPN. These diseases are characterized by thrombohemorrhagic complications and, as with MDS, are associated with a risk of transformation to AML [36]. Although the disease manifestations can be different, the subtypes can share common driver mutations in *JAK2, CALR* and *MPL* [36]. The risk of thrombotic complications can be reduced by low-dose aspirin treatment or by cytoreductive therapy using hydroxyurea, interferon alfa or peginterferon alfa [36]. As novel agents become available, care for individuals with advanced MPNs such as myelofibrosis may improve.

The final group of HMs in our analysis was MM and the CMLs (see first paragraph of Discussion: survival in CML was somewhat underestimated). Both show a two-phase development in survival, an initial phase of slow development (lasting until about 2004 in MM and to 2000 in the CMLs) which was followed by a rapid improvement. The development of treatment for MM is described in detail elsewhere [37]. Magnetic resonance imaging has become an important tool for the characterization of bone lesions. The main changes in SE included adoption of high-dose melphalan and HSCT from the late 1980s, and from the early 2000, vincristine, adriamycin, and dexamethasone as induction treatment. Agents with novel mechanisms of action were then employed, including immunomodulatory agents (e.g., thalidomide, lenalidomide) and proteasome inhibitors (e.g., bortezomib) which have transformed the management of MM and coincide with large improvements in relative survival (2005–2014). In SE from the year 2010, bortezomib and thalidomide became part of standard induction therapy [38]. According to the data from 2008, only 31% of the MM patients had received thalidomide, bortezomib or lenalidomide but the proportion increased to 68% by 2012 [38]. These therapeutic changes were considered important in boosting survival in USA [39, 40]. An earlier SE study emphasized the beneficial effects of autologous HSCT [41]. Novel proteasome inhibitors and immunomodulatory agents have been introduced, and more recently immunotherapies have become available. CML is characterized by the t(9;22) chromosomal abnormality resulting in a BCR::ABL1 fusion protein [42]. Before the 1980s treatment consisted of an alkylating agent with hydroxyurea, while HSCT with interferon alfa was used soon after [42]. In 2000 the first tyrosine kinase inhibitor, imatinib mesylate was introduced specifically targeting the BCR::ABL1 oncoprotein and blocking its action. It has revolutionized the treatment of CML and serves as a paradigm for targeted therapy in oncology [21]. Its use was started in SE in 2001 and was associated with remarkable improvements in survival [42].

The first analyses of NORDCAN data for HMs concluded that DK survival data were consistently below those of other countries [9]. The present NORDCAN analysis confirms inferior survival of HMs in DK in the 1970s, particularly for MM, CLL and CML. However recent data suggest a large improvement in relative survival in DK, which started towards the end of the 1990s, resulting in DK being ranked best in 1- and 5-year survival in 2015–2019 in most of the HMs. FI showed modest development, with a notably low improvement in relative survival over the past 10 years. Finland experienced a deep economic crisis at the beginning of 1990s from which recovery was slow; in 2011 purchase power corrected health expenditure per capita was $3374 in FI, while in NO, DK and SE it was $5669, 4448 and 3925 (https://www.oecd.org/els/health-systems/Health-at-a-Glance-2013.pdf). Funding may be one of many explanations as we show a discrepancy between NO expenditure and survival.

The NORDCAN database uses the ICD-10 classification for cancer types which for some HMs, such as NHL and ALL limits analysis because of diverse subtypes. Even for other HMs, it is not possible to specify the exact disease subtype, which may have distinct risk and survival profiles. NORDCAN is lacking information on disease-specific stage, which is an important determinant of survival. Treatment information is also lacking and we therefore only discuss the general trends in treatment using data from the literature or, for newer medication, from approval dates of the Swedish or the European medicines authorities. Even with such weaknesses, NORDCAN is the only database that offers high-quality nation-wide cancer data for over a half century.

In conclusion, the long-term survival data correlated with the prevailing treatment approaches allowed a visual assessment of the possible factors influencing 5-year survival. We need to acknowledge our inability to assess the positive role of earlier diagnosis and in general diagnostic improvements, techniques of disease monitoring, such as minimal residual disease, control of infections and other comorbidities, as well as optimization of patient care. For HMs, HL and CLL, reaching high 5-year survival (80–95%), the increase has been steady and almost linear suggesting that an optimized use of the existing therapeutic options and other patient care enabled the success. Increase in survival for MPN has also been steady, as it has been for MDS at a lower survival level and with large country-specific differences. Survival curves for MM and the CMLs showed a strong upward curvature following the use of novel medication, proteasome inhibitors and immunomodulators in the case of MM and imatinib in the case of CML. Survival in AML, which was <5% 50 years ago, has increased to 20 or 30% with the use of chemotherapy agents. However, a number of novel therapies have been introduced recently with aim of significantly improving survival rates over the next decade. Despite such advances, our analysis highlights patients diagnosed with an HM > 70 years of age as an area of unmet need. As these patients account for half of all patients for most HMs, advances in population-level survival are dependent on improvements in care for these older patients.

## Supplementary information

Supplementary Table 1

## Data Availability

Publicly available data were used from the NORDCAN database.
